# Plasmonic Metasurface Absorber Based on Electro-Optic Substrate for Energy Harvesting

**DOI:** 10.3390/ma11112315

**Published:** 2018-11-18

**Authors:** Naseer Muhammad, Tao Fu, Qiang Liu, Xiaopin Tang, Zi-Lan Deng, Zhengbiao Ouyang

**Affiliations:** 1 THz Technical Research Center of Shenzhen University, Shenzhen Key Laboratory of Micro-Nano Photonic Information Technology, Shenzhen 518060, China; naseer@szu.edu.cn (N.M.); qliu@szu.edu.cn (Q.L.); xptang@szu.edu.cn (X.T.); 2 Key Laboratory of Optoelectronics Devices and Systems of Ministry of Education and Guangdong Province, Shenzhen 518060, China; 3 College of Electronic Science and Technology, Shenzhen University, Shenzhen 518060, China; 4 Guangxi Key Laboratory of Precision Navigation Technology and Application, Guilin University of Electronic Technology, Guilin 541004, China; ft85@guet.edu.cn; 5 Guangdong Provincial Key Laboratory of Optical Fiber Sensing and Communications, Guangzhou 510632, China; zilandeng@jnu.edu.cn; 6 Institute of Photonics Technology, Jinan University, Guangzhou 510632, China

**Keywords:** metasurface, absorption, energy, plasmon, electro-optic

## Abstract

A highly efficient and broad light absorber capable of wide-angle absorption in the visible and near infrared range is presented and numerically investigated for energy harvesting in a simple geometry. According to the calculated results, the proposed device has a peak absorption level of about 99.95%. The actual absorption efficiency is 76.35%, which is approaching that of complex multilayer absorbers with 88 layers working in the wavelength range of 300 nm to 2000 nm. The electro-optic material has the potential of shifting the absorption peak position, compensating fabrication errors and thus reducing the fabrication technique difficulties. Also, the high electro-optic tunability can be used for filters, infrared detection, and imaging applications. More directly, the proposed absorber can be potentially deployed in solar cells and solar thermals.

## 1. Introduction

Plasmonic metamaterials composed of subwavelength metallic structure cells arranged in periodic arrays, demonstrate exotic optical properties such as negative refractive index [1,2], near zero index [3] superlens [4], Fano resonances [5,6,7] and high field localization. Perfect absorption, an important functionality related to field localization, has attracted extensive attention due to applications in energy harvesting [8,9], light emitting diodes [10,11], sensing [12,13,14,15], and optical filters [16]. Plasmonic metamaterials possess high intrinsic losses, which can be increased significantly to achieve perfect absorption by proper engineering the structures. Since the first perfect absorber was designed in 2008 [17], different designs have been proposed to absorb specific regimes of electromagnetic waves, including visible [18], infrared [19], terahertz [20], and gigahertz [21,22,23]. Typically, these designs consist of three layers: a top layer, a dielectric spacer, and a metallic substrate, resulting in a metallic sub-wavelength resonator structure [24,25]. The absorption capability strongly depends on the geometric configurations of the design. The optical and electrical behavior of the plasmonic absorber can be tuned by varying these configurations [11]. Photovoltaic cells use absorption and re-emission mechanism to convert solar energy into electrical energy. Therefore, to increase the efficiency of the photovoltaic cells, the plasmonic absorbers need to absorb solar radiation in a wide waveband [26]. In perfect absorbers, narrow resonances are suitable for applications like sensors [15]. However, for solar energy harvesting, broad resonances in the visible-near-infrared regime and polarization insensitivity are most important. To increase the spectral width of absorption, one needs to induce multiple resonances in absorption spectra [25,26,27,28,29,30]. Habib et al. proposed a multiband absorber, the absorption covers almost the visible range, only at 50% absorption level [30]. X. He et al. reported a highly broad-band absorber (284–1524 nm) with an average absorption level of 92%; however, the absorption level was not maintained above 30 degrees of polarization [31]. M. Batuhan and S. Cumali investigated a polarization-independent multiband absorber; however, the largest resonance width was only 34 nm at 99.4% absorption level [26]. Nielsen et al. reported perfect absorption at an average level of 94% in the visible range, but the structure was incident angle-dependent [32]. P. Rufangura and C. Sabah reported a dual-band absorber for solar cells; however, the spectral widths of resonances were narrow [33]. Zhu et al. proposed a very broad polarization-insensitive absorber for solar energy harvesting; however, this design was a stack of about 44 pairs of Gold/Silicon alternating layers [34], making the fabrication process very complicated. Lei et al. proposed a tri-layer absorber with a relatively thin geometry; however, the design could absorb shorter wavelengths of up to only 1200 nm, and it shows limitations in terms of incident angle, and lacked wavelength tunability and geometric flexibility [35]. Hedayati et al. reported a very interesting perfect absorber, although the structure lacked wavelength tunability and the absorption was limited to visible frequencies [36]. Wu et al. proposed a broad, perfect absorber, but it was polarization- and angle-dependent [37]. Chen et al. demonstrated a very interesting broadband absorption; however, it showed polarization and incident angle dependencies [38]. Yan et al. experimentally achieved broadband absorption in random nanoparticles, although for production, the exact geometric parameters are important, as their simulation results suffered from the same problem [39]. In summary, the challenge was that the reported absorber structures were polarization-dependent [31,37,40], angle-dependent [31,35,38,41], were for selective wavelengths [42,43], or small wavebands [22,26,41,44], or suffered from narrow spectral width [26,30,42,44,45,46,47,48], or lacked wavelength tunability and geometric flexibility [31,34,37,39], or required structure scaling for wavelength adjustment, which can increase the use of materials, time consumption and production cost. To overcome these problems, one needs to design wide-angle, few-layer highly tunable absorbers, to absorb the wavelength range of 300 nm to 2000 nm and above. 

In this article, we propose a tri-layer absorber with multispectral broadband in a wide range of incident angle. Highly broad resonant peaks are achieved in the visible and near infrared regime. Distinct from previous work reported in [21,30,49,50], our suggested design consists of a relatively simple geometry of split square rings and disk with an electro-optic dielectric layer, exhibiting outstanding absorption efficiency having less incident-angle dependence in wide-angle range, and wavelengths tunability. The spectral width at the absorption level of 90% is 404 nm. The total absorption efficiency (actual absorption efficiency in a wide waveband, different from the absorption efficiency in a narrow band) is found to be 76.35% in the waveband of 500 nm to 2300 nm by applying the integration to the solar intensity spectrum distribution absorbed by our design which gives us the exact absorption efficiency. The 76.35% efficiency is approaching the high actual absorption efficiency of the complex multilayer absorber with 88 layers [34] working in the broad solar spectrum from 300 nm to 2000 nm. The absorber performance is evaluated by changing the geometric parameters, and shifted by input voltage to reduce the fabrication errors. The wide range of incident angle and the broad wavelength range are more suitable for solar energy harvesting and solar thermals [8]. Moreover, the wavelength shift based on electro-optic effect, which is different from the thermal tunability [42], demonstrates promising prospects for infrared detection and imaging.

## 2. Physical Design

**Figure 1:**
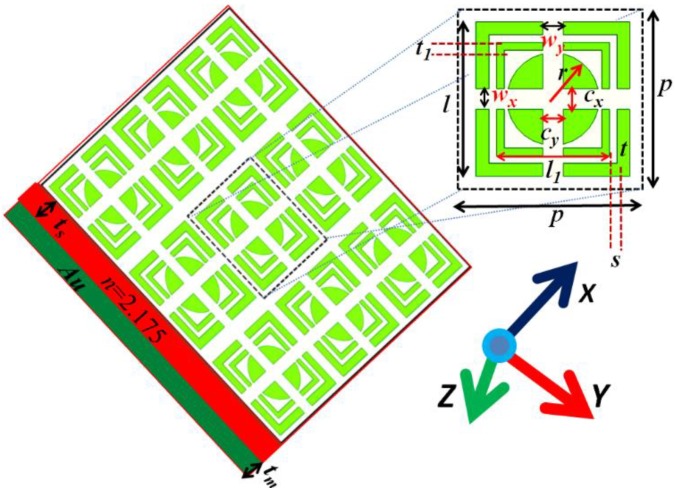
Schematic of the three-dimensional absorber.

The proposed absorber has three layers: a layer of a periodic array of square unit cells made of metallic split square rings and disks, a layer of electro-optical material, and a metallic substrate, as shown in Figure 1. The side length of the square unit cell is 400 nm. The other geometric parameters are: the side length of each outer square ring is l= 300 nm, the side length of each inner square ring is $l_{1}=l-80$ nm, the horizontal and vertical splits in square rings and disk are represented by *w_x_*, *w_y_* and *c_x_*, *c_y_*, whose optimum values are to be found in simulations, the resonator thickness in −*z* to *z*-direction is 30 nm, the space *s* between the square rings is 15 nm, radius of the disk is r= 75 nm, thickness of outer ring t= 25 nm and inner ring $t_{1}=$ 15 nm, the thickness of the electro-optical dielectric layer is $t_{s}=70\;$ nm, which is sufficient for absorption at shorter wavelengths where the role of metallic part is more important (plasmon decay and Ohmic losses) [51], the thickness of the metallic substrate used as reflector to reduce the transmission (approximately 0) is $t_{s}=730$ nm, *θ* is the incident field angle, and $V_{0}$ is the applied voltage, respectively. In the following, except for different applied voltage case, which shows the tuning property, the applied voltage is fixed as $V_{0}=1$ V for all other simulations. Lithium tantalate (LiTaO_3_) is used as a suitable electro-optic material, which fine-tunes the dielectric environment to the resonator in the absorber. The varied refractive index n of the electro-optical material due to the Pockels effect by applying an external electric field unlike thermal tuning [42] can be calculated by the formula $n=n_{0}+0.5n_{0}^{3}\gamma E_{ex}$ [16]. The electro-optic coefficient (eoc) γ= 8 pm/V is considered. The external electric field is $E_{ex}=V_{0}/t_{s}$. The ordinary refractive index of Lithium tantalate is $n_{0}=2.175$. The ordinary optical axis of Lithium tantalate is set in the *x*-axis (electric field in *x*-axis at 0°, unlike selective incident angle [42,43]), and the extraordinary optical axis in the *z*-direction [16], so that the electric fields of the waves are perpendicular to the extraordinary optical axis, and thus only the ordinary refractive index is to be considered in the simulations. Moreover, the electric vector of the incident wave is always normal to the extraordinary axis, so the Lithium tantalate layer will behave the same for the different incident angle of the waves, which will add to less dependence of optical property on the incident angle. Above the electro-optical layer, the metallic rings and disk are in the air. Drude-Lorentz dispersion model has been used for the dielectric constant of the gold (the substrate reflector) having plasma frequency of 1.37 × 10^16^ (rad/s) [16] in the waveband of 500 nm to 2300 nm. However, for shorter wavelengths, one may use a more advanced model, such as the Drude model with two critical points [52]. 

The solar energy spectrum has a non-uniform distribution in a wide range of wavelengths. To calculate the exact absorption efficiency, also called actual absorption efficiency or total absorption efficiency, of our designed structure we apply the efficiency integration formula: $\eta_{T}=$ ∫I(λ)A(λ)dλ/∫I(λ)dλ [38], where I(λ) is the solar intensity spectrum distribution, A(λ)=1−R(λ) [53] is the absorption coefficient spectrum of the device because the transmitted wave to the bottom under the metallic substrate can be regarded as zero, and R(λ) is the reflectivity proportional to the square of the electric field of the reflected wave. All the computations are carried out by COMSOL Multiphysics software (4.4, Shenzhen University, Shenzhen, China) with RF module. The simulation area is covered by Floquet periodic boundary conditions, which repeat the unit cells both in *x*- and *y*-directions.

## 3. Results and Discussion

### 3.1. Basic Studies

In the structure, the top layer, consisting of the metallic resonator, is responsible for the line-shaping of the optical response. The middle layer is an electro-optic spacer acting as the central interaction region of an anti-Fabry-Perot cavity whose upper and lower mirrors are, respectively, the top layer and the metallic substrate. The proposed structure has five advantages: (a) multiple resonances, (b) flat-resonant peaks highly suitable for broadband optical filtering and detection, (c) resonances are in the visible and infrared range which are promising for energy harvesting, (d) the resonances are tunable to the desired range with variation in the applied voltage and dopants of substrate, and (e) efficient at different substrate materials. The substrate provides a path for repeated-reflection resonances, leading to resonance absorption in the substrate. However, the pattern of the metasurface decides the details of absorption, e.g., wavelength and peaks. Moreover, absorption also exists in the metasurface, but it was much lower than that in the substrate, because the substrate has a much larger absorption volume than the metasurface. The bottom thick metallic substrate (Au) has a thickness larger than the penetration depth of the electromagnetic waves (opaque medium); in turn, this results in zero transmission (T(λ)=0), thus A(λ)=1−R(λ) or $A\left(\lambda \right)=\left(1-{\left|S_{11}\right|}^{2}\right)$ [22,23,53]. 

**Figure 2:**
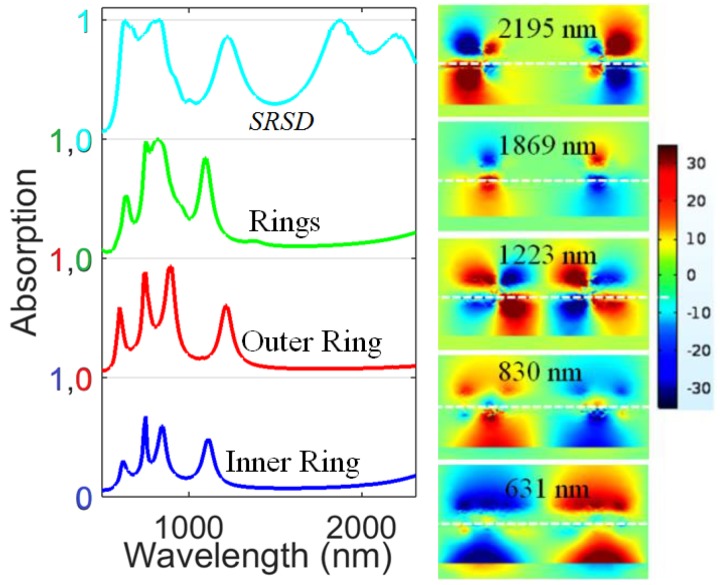
Absorption spectra of the inner ring, outer ring, two rings, and split rings + split disk (SRSD). The inset shows the electric field distribution of light normalized to the incident field at resonant wavelengths in the *zx*-plane intersecting the center of unit cell (y=0) for the SRSD structure. The white dotted line demonstrates the interface of resonator and substrate.

To better analyze the absorption properties of the absorber, first of all, we consider the case that there is only the thin inner ring, without splits. The thin inner ring results in four resonant peaks, as shown in Figure 2 (blue). These peaks emerge due to the excitation of the resonant modes of three layers; however, more light is reflected to space [54]. To check the validity of the simulated result, we carried out the following analysis. In resonance, the optical circumference of the ring should be $L_{oc}=nL_{c}=p\lambda /2$, where $L_{c}$ is the circumference of the ring, *p* is an integer, and λ is the resonance wavelength of the ring. The optical circumference of the ring is $L_{c1}=4\left(l_{1}-t_{1}\right)$ for the inner ring and $L_{c1}=4\left(l-t\right)$ for the outer ring. 

With the operating parameters for Figure 2, we can obtain the following resonance wavelength in the inner ring: $\lambda =2nl_{c1}/p=\;$(1189.321 nm (p=3), 891.991 nm (p=4), 713.593 nm (p=5), 594.661 nm (p=6)), which agrees well with the resonance absorption peak wavelengths obtained from the simulation result (1112 nm, 841 nm, 750 nm, 620 nm) shown in Figure 2. The simulated resonance absorption peak wavelengths are a little less than that obtained by estimation due to the fact that there are large numbers of rings on the metasurface, and the rings are coupled together.

Next, we consider the case in which there is only the outer ring, which has a larger size and an obviously larger effective refractive index than the inner ring, due to which the absorption peaks shift to longer wavelengths, as shown in Figure 2 (red). The absorption levels at all peaks are higher than that due to the inner ring, because the larger ring has a larger absorption volume than the inner ring, leading to more absorption in the metasurface, decreasing reflection. In the same way as for the analysis of the inner ring, the resonance wavelength on the outer ring by the estimation is $\lambda =2nl_{c2}/p=$ (1196.573 nm (p=4), 957.259 nm (p=5), 797.716 nm (p=6), 598.287 (p=8)), which also agrees well with the resonance absorption peak wavelengths obtained from the simulation results (1212 nm, 891 nm, 750 nm, 600 nm) shown in Figure 2. Here, a suitable integer *p* is to be chosen, because the resonances are influenced by the structure under the rings.

Furthermore, we consider the case in which both the inner ring and outer ring are present. The absorption peaks again increase, as shown in Figure 2 (green), due to the increased absorption volume of the metasurface. A broader band of absorption appears due to the collaboration of more resonant modes originating from the inner ring and outer rings in that band. Finally, we consider the split-rings + split-disk (SRSD) structure, where splits are introduced in the resonator to decrease the amplitude of reflection [30]. The splits in the horizontal and vertical directions are *w_y_* = 10 nm, *c_y_* = 0 nm and *w_x_* = *c_x_* = 20 nm, respectively. We obtain broad absorption spectra, as shown in Figure 2 (sky blue), due to light localization in the splits, which decrease the reflection, as reported in [30]. Furthermore, due to the splits, even more modes can exist in the structure, and thus a wider absorption band can exist. At shorter wavelengths, the role of the metallic part is more important because of the high Ohmic losses and plasmon decay [51]. Moreover, due to the disks, absorption peaks in the longer wavelength region appears, as the disks have a larger effective refractive index. The amplitude of the reflection decreases with multiple splits in the standalone top layer [30]. The inset shows the electric field distribution of light in the structure in the *zx*-plane normalized to the incident light wave electric field for the SRSD structure. It can be seen that the splits strongly couple the incident beam to the substrate, which localizes the electric field. The strength of the localized electric field seems stronger at the metal-dielectric-metal interfaces, results in broadband absorption.

For solar cell applications, as the light is mainly absorbed by the metallic substrate, so a P-type semiconductor material can be set below the substrate. Without incident light, some free electrons in the substrate can diffuse into the semiconductor region, leading to a PN junction. When there is incident light, more free electrons will be generated, or higher energy of free electrons will be obtained in the substrate, so that more electrons can enter the semiconductor region, forming a current and generating electric power when a resistor load is connected between the substrate and the semiconductor.

### 3.2. Influence of Structural Parameters on the Absorption Spectra

In this section, we study the impact of structural parameters on the absorption spectra of the SRSD absorber. First, we vary the horizontal split *w_x_* from *w_x_* = 20 nm to 60 nm while fixing *c_x_* = 20 nm, *w_y_* = 10 nm, and *c_y_* = 0 nm. A maximum total absorption is found to be 62.86% at *w_x_* = 50 nm, as seen in Table 1. 

Next, we fix *w_x_* = 50 nm, *c_x_* = 20 nm, and *c_y_* = 0 nm to study the influence of *w_y_* further. For this case, maximum total efficiency is found to be 62.06% at *w_y_* = 20 nm, as shown in Table 1. Now we consider the SRSD structure with the following intermediate optimum values: *w_x_* = 50 nm, *w_y_* = 20 nm, and *c_x_* = 20 nm, but *c_y_* varying from 20 nm to 60 nm. The maximum value of total efficiency, calculated at *c_y_* = 20 nm, is 66.90%, as shown in Table 1. This shows that, with an increase in split size *c_y_* from 20 nm to 60 nm, the total efficiency decreases.

Furthermore, we move to consider the full structure to see the influence of the combined split on the absorption, where *w_x_*= *w_y_*= *c_x_*= *c_y_* = *CS* is varied from 20 nm to 60 nm. Here, we consider different radii of the spherical disk from *r* **=** 75 nm to *r* = 80 nm for the SRSD structure.

**Figure 3:**
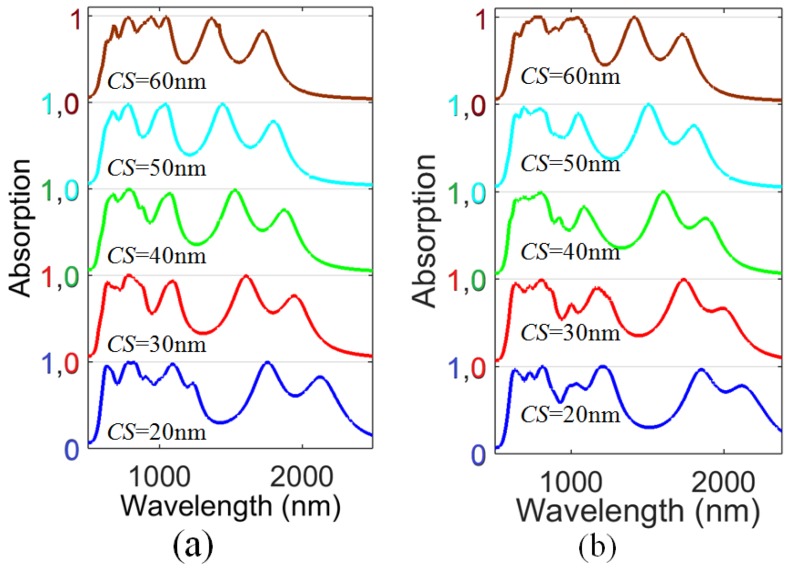
Absorption spectra for different combine split (*CS*) sizes at (**a**) *r* = 80 nm, and (**b**) *r* = 90 nm.

The results are shown in Figure 3 and Table 2. The spectral width of the absorption increases due to the decrease in the dips between the first three resonances. The other two broad resonances shift to shorter wavelengths (the higher solar intensity region), as shown in Figure 3a, promoting the absorption efficiency of our design in the visible and near infrared region. This shift is due to the increase in *CS*, which results in a decrease of the effective size of the resonator and thus shorter resonance wavelength. The total absorption efficiency (the actual absorption efficiency) is 69.98%, calculated for *CS* = 20 nm, as shown on the first line in Table 2. Furthermore, we increase the radius *r* of the spherical disk to *r* = 90 nm and vary the *CS* from 20 nm to *CS* = 60 nm, as shown in Figure 3b and on the second line in Table 2. At *CS* = 60 nm, the absorption peaks of the resonances become highly broad, and the spectral width reaches 75 nm (742 nm–817 nm), 90 nm (972 nm–1062 nm) and 40 nm (1392–1432nm) at an absorption level of 97%. From Table 2, it can be seen that at *CS* = 60 nm a small increase in efficiency occurs. This is due to the fact that the modulation depths around 1173 nm (*r* = 80 nm) and 1222 nm (*r* = 90 nm) enormously decreases compared to *CS* = 50 nm, as the two resonant modes strongly shift to shorter wavelengths. As *CS* increases, the absorption spectrum shifts towards the short-wavelength region. In a certain band, the shorter region means higher solar strength and more energy absorbed, but in some band, the solar radiation strength on earth surface may decrease. This may be responsible for the small increase in the total absorption efficiency at *CS* = 60 nm.

It is important to note that the peak absorptions at these two resonances are 99.8% and 99.4% at around 762 nm and 1041 nm, respectively. The third resonant peak absorption level is 99.6% at around 1417 nm, as shown in Figure 3b. The structure possesses total absorption efficiency of 69.22% at *CS* = 20 nm, as shown in Table 2.

To see the influence of the substrate, we considered the *CS* = 60 nm spectrum, due to its broad flat peaks and high absorption level, as can be seen in Figure 3b, and applied different materials and varied the thickness from 70 nm to 110 nm. The efficiency of the absorber decreases with an increase in the thickness of the substrate, as shown in Table 3.

The highest efficiency value was 76.35%, calculated for AZO substrate, and approaching the absorption efficiency of complex multilayer absorbers with 88 layers working in a wavelength range of 300 nm to 2000 nm [34].

**Figure 4:**
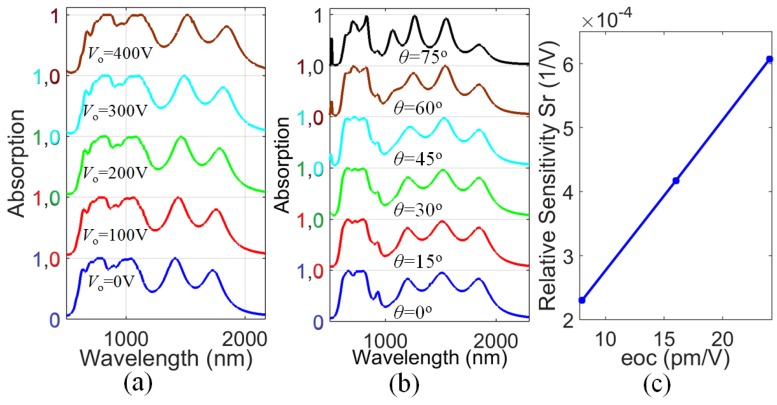
(**a**) Absorption spectra of different applied voltages *V*_0_ at *CS* = 60 nm and *r* = 90 nm; (**b**) influence of incident angle *θ* on absorption, and (**c**) relative sensitivity at different eoc values.

From Figure 3b, it can be seen that the *CS* = 60 nm spectrum has broad flat peaks and a high absorption level; therefore, we select *CS* = 60 nm and calculate the absorption for different applied voltages $V_{0}$ in the range of 0 to 400 V, with a step interval of 100 V, as shown in Figure 4a. The refractive index of the electro-optic substrate varies with an increase in applied voltage $V_{0}$. The resonant wavelengths are very sensitive to the change in the background materials. The resonant modes shift from 635, 795, 1041, 1412, and 1728 nm to longer wavelengths 675, 871, 1127, 1518, and 1843 nm, respectively. This is so because the refractive index increases as the voltage increases [16], and thus the resonant wavelengths become longer. The absorption spectra are shifted by applying different applied voltages without changing the spectral profile, which is distinct from thermal tunability [42]. This voltage-based sensitivity can be a promising parameter for broadband optical filtering. The relative sensitivity $S_{r}$ can be calculated by $S_{r}=\left(\Delta \lambda /\lambda \right)/\Delta V_{0}$, where Δλ is the change in resonant wavelength, λ is an average wavelength in the band of observation, and $\Delta V_{0}$ is the change in the applied voltage. The highest relative sensitivity of 2.3 × 10^−4^ V^−1^ is calculated at the resonant mode around 795 nm, which shifts to 871 nm. This resonance shift can be useful for adjusting the resonant wavelengths to the range of interest to reduce fabrication errors. Figure 4c shows relative sensitivity at different γ (pm/V) values. γ is increased from 8 pm/V to 24 pm/V with a step size of 8 pm/V. As γ increases, the relative sensitivity increases from 2.3 × 10^−4^ V^−1^ to 6.07 × 10^−4^ V^−1^. However, the efficiency of the absorber decreases as we increase the applied voltage. This is because as we increase the applied voltage, the resonant wavelengths shift to the low solar intensity regime.

To observe the influence of incident beam angle *θ* on the absorption of the designed structure, absorption spectra under different angles with a solar hour angle step interval of 15° are shown in Figure 4b. It is found that our design shows high absorption at all angles. From the design outcomes, it can be said that the absorber illustrates the expected results and can have wide-incident-angle applications. From the results, we can see that the structure can absorb a wide range of visible and near-infrared waves, so it can serve as a wideband band-stop filter with tunability. Also, it can be used in mid-infrared cameras for eliminating undesired visible and near infrared background interfering waves.

## 4. Conclusion

We numerically investigated a new absorber with multi broadband capability within a wide range of incident angles. Our design is based on an electro-optic substrate, and thus the absorption band can be shifted by the applied voltage. The single resonance absorption band in the multispectral absorber was investigated up to 107 and 44 nm, with absorption levels of 97 and 99.75%. In comparison, the spectral width of 1023 nm at an absorption level of 70% in our design is higher than that in previously reported broad-resonance tri-layer plasmonic perfect absorbers [25,26,30,44,45,46,47,48,54] for solar cells. The total absorption efficiency was calculated to be 76.35% in the band from 500 nm to 2300 nm. This efficiency approaches the high actual absorption efficiency values calculated in complex multilayer absorbers of 88 layers working in the wave-band from 300 nm to 2000 nm and above. These deep subwavelength structures, artificially engineered for extraordinary control and manipulations of light, can be fabricated by advanced techniques that are able to fabricate very small structures down to few nanometers [55]. To achieve high absorption efficiency, different designs from simple to complex configurations can be explored. The proposed structure has high geometric flexibility and applied voltage-based resonance shifting potential. Thus, it can provide a suitable plat-form for outstanding detectors and broadband optical filters.

## Figures and Tables

**Table 1:** Total efficiency $\eta_{T}$ at different split sizes.

Parameters	Split	20 nm	30 nm	40 nm	50 nm	60 nm
*w_x_*	$\eta_{T}$ (%)	61.28	62.02	62.51	62.86	62.36
*w_y_*	$\eta_{T}$ (%)	62.06	61.29	61.70	61.14	62.04
*c_y_*	$\eta_{T}$ (%)	66.90	66.31	65.33	63.59	62.31

**Table 2:** Total efficiency $\eta_{T}$ at different split sizes.

Disk Radius	*CS*	20 nm	30 nm	40 nm	50 nm	60 nm
*r* = 80 nm	$\eta_{T}$ (%)	69.98	66.94	65.27	61.68	63.99
*r* = 90 nm	$\eta_{T}$ (%)	69.22	67.85	65.30	63.44	66.96

**Table 3:** Total efficiency $\eta_{T}$ at different substrate thickness and materials.

Substrate Thickness	$\;t_{s}\;$	70 nm	80 nm	90 nm	100 nm	110 nm
AZO	$\eta_{T}$ (%)	76.35	67.97	65.26	57.17	50.36
ITO	$\eta_{T}$ (%)	75.31	66.40	62.78	55.01	48.33
SiO_2_	$\eta_{T}$ (%)	70.36	67.94	68.76	64.61	60.20
ZnO	$\eta_{T}$ (%)	72.96	63.93	60.26	51.62	46.56
